# Auxin regulation and *MdPIN* expression during adventitious root initiation in apple cuttings

**DOI:** 10.1038/s41438-020-00364-3

**Published:** 2020-09-01

**Authors:** Ling Guan, Yingjun Li, Kaihui Huang, Zong-Ming (Max) Cheng

**Affiliations:** 1 College of Horticulture, Nanjing Agricultural University, 210095 Nanjing, China; 2 Institute of Pomology, Jiangsu Academy of Agricultural Sciences, Jiangsu Key Laboratory for Horticultural Crop Genetic Improvement, 210014 Nanjing, China; 3 Department of Plant Sciences, University of Tennessee, Knoxville, TN 37831 USA

**Keywords:** Non-model organisms, Transcriptional regulatory elements

## Abstract

Adventitious root (AR) formation is critical for the efficient propagation of elite horticultural and forestry crops. Despite decades of research, the cellular processes and molecular mechanisms underlying AR induction in woody plants remain obscure. We examined the details of AR formation in apple (*Malus domestica*) M.9 rootstock, the most widely used dwarf rootstock for intensive production, and investigated the role of polar auxin transport in postembryonic organogenesis. AR formation begins with a series of founder cell divisions and elongation of the interfascicular cambium adjacent to vascular tissues. This process is associated with a relatively high indole acetic acid (IAA) content and hydrolysis of starch grains. Exogenous auxin treatment promoted this cell division, as well as the proliferation and reorganization of the endoplasmic reticulum and Golgi membrane. In contrast, treatment with the auxin transport inhibitor *N*-1-naphthylphthalamic acid (NPA) inhibited cell division in the basal region of the cuttings and resulted in abnormal cell divisions during the early stage of AR formation. In addition, PIN-FORMED (PIN) transcripts were differentially expressed throughout the whole AR development process. We also detected upregulation of *MdPIN8* and *MdPIN10* during induction; upregulation of *MdPIN4*, *MdPIN5*, and *MdPIN8* during extension; and upregulation of all *MdPIN*s during AR initiation. This research provides an improved understanding of the cellular and molecular underpinnings of the AR process in woody plants.

## Introduction

Propagation via cuttings is the most economical method for the mass production of horticultural and forestry plants while simultaneously maintaining desirable genetic traits^1,2^. Adventitious root (AR) formation is essential to this process, and AR induction has been studied for decades^3,4^. AR formation is affected by many factors: juvenility; ontology; species/genotype; various environmental conditions, such as extreme temperatures and salt stress; the content of H_2_O_2_, NO, and Ca^2+^; and endogenous and exogenous applications of plant hormones^5,6^. Although AR formation has been studied at the anatomical level, its molecular basis, including the mechanism that triggers the process, remains unclear^7^. AR formation can be divided into three phases: induction, initiation, and extension, which lead to new visible root systems. ARs can initiate from internodes, callus formed at the base of cuttings, or the hypocotyl of herbaceous plants^8,9^. In apple, ARs emerge from lenticels, in which large intracellular spaces allow for gas exchange in the stems.

According to recent anatomical studies in *Arabidopsis thaliana*, founder cell division gives rise to AR primordia (ARP), which then develop into new roots^10^. Similar to that of primary roots or lateral roots (LRs), the indeterminate growth of ARs depends on cell division and elongation, which are affected by various environmental conditions^11^. AR formation is regulated by nearly all the major plant hormones, with auxin strongly promoting this process^12^. Exogenous auxin treatment or elevated endogenous auxin levels through genetic engineering increase the rate of AR formation and the number of ARs formed, whereas impaired auxin signaling or transport via mutagenesis or auxin transport inhibitors inhibit AR initiation^13,14^.

The auxin gradient acts as a master controller of AR formation, development, and geotropic responses^15,16^. The relationship between the postembryonic roots of AR (and LR) formation and polar auxin transport (PAT) was established by using the auxin transport inhibitor 1-*N*-naphthylphthalamic acid (NPA)^17,18^. The promoting effect of PAT on AR development can be counteracted by NPA, which disrupts PAT and, consequently, AR formation^19^.

The asymmetric cellular localization of PIN-FORMED (PIN) auxin efflux carriers has been shown to play a rate-limiting role^20^ in directing cell-to-cell auxin flow^21,22^. Despite their importance, the roles of auxin and PINs in regulating this process in woody horticultural plants have rarely been studied. Here, we integrated anatomical and ultrastructural observations, hormone analysis, and the spatiotemporal expression profile of the *PIN* genes to investigate the details of early initiation and organization of ARP within the lenticels of apple (*Malus domestica*) rootstock M.9 cuttings.

## Results

### AR initiation in apple cuttings

**Fig. 1:**
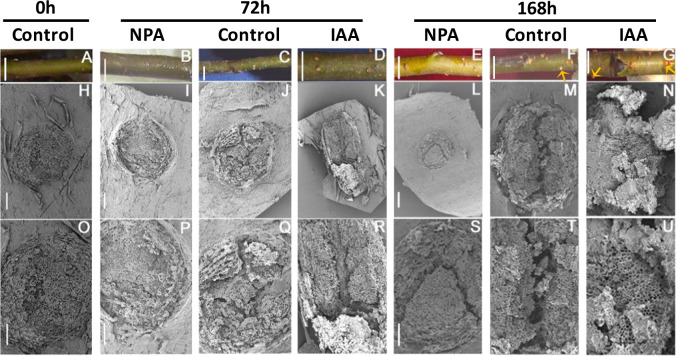
Morphological changes in lenticels upon NPA and IAA treatments. Apple cuttings were cultured in Hoagland’s solution and sampled every 24 hours (h) from 0 to 168 h. Three distinctive development phases were observed at 0, 72, and 168 h. **a**–**g** Physical appearance of submerged cuttings. Arrows point to the origination of new ARs. **h**–**u** SEM micrographs of lenticels at different AR developmental phases; Bars: **a**–**g** = 0.4 cm, **h**–**u** = 100 μm. Bars **i** = **j** = **k**; **l** = **m** = **n**; **p** = **q** = **r**; and **s** = **t** = **u**.

To determine the exact position of AR formation, 6-month-old M.9 apple cuttings were cultured in Hoagland’s nutrient solution. The submerged stems were evaluated for their AR formation over a 7-day period (168 h), during which the lenticels continuously expanded (Fig. 1a, c and f). Noticeable lenticel enlargement was observed at 72 h, and new ARs emerging from the lenticels were easily observed as small protrusions at 168 h (Fig. 1f). Scanning electron microscopy (SEM) analysis revealed progressive longitudinal splitting of the submerged lenticels in the controls until a deep fissure was observed at 168 h (Fig. 1h, j, m, o, q and t). Indole acetic acid (IAA) treatment accelerated the longitudinal splitting of the lenticels at each time point. For example, compared with that of the corresponding controls, the lenticel dehiscence in the NPA-treated cuttings became deeper at 72 h (Fig. 1k, r), and the lenticel surface was nearly completely disrupted at 168 h (Fig. 1n, u). By contrast, lenticel longitudinal splitting was very thin and shallow in NPA-treated cuttings at 72 h (Fig. 1i, p), and the fissures were still very narrow and shallow at 168 h compared to those of the control cuttings (Fig. 1l, s).

**Fig. 2:**
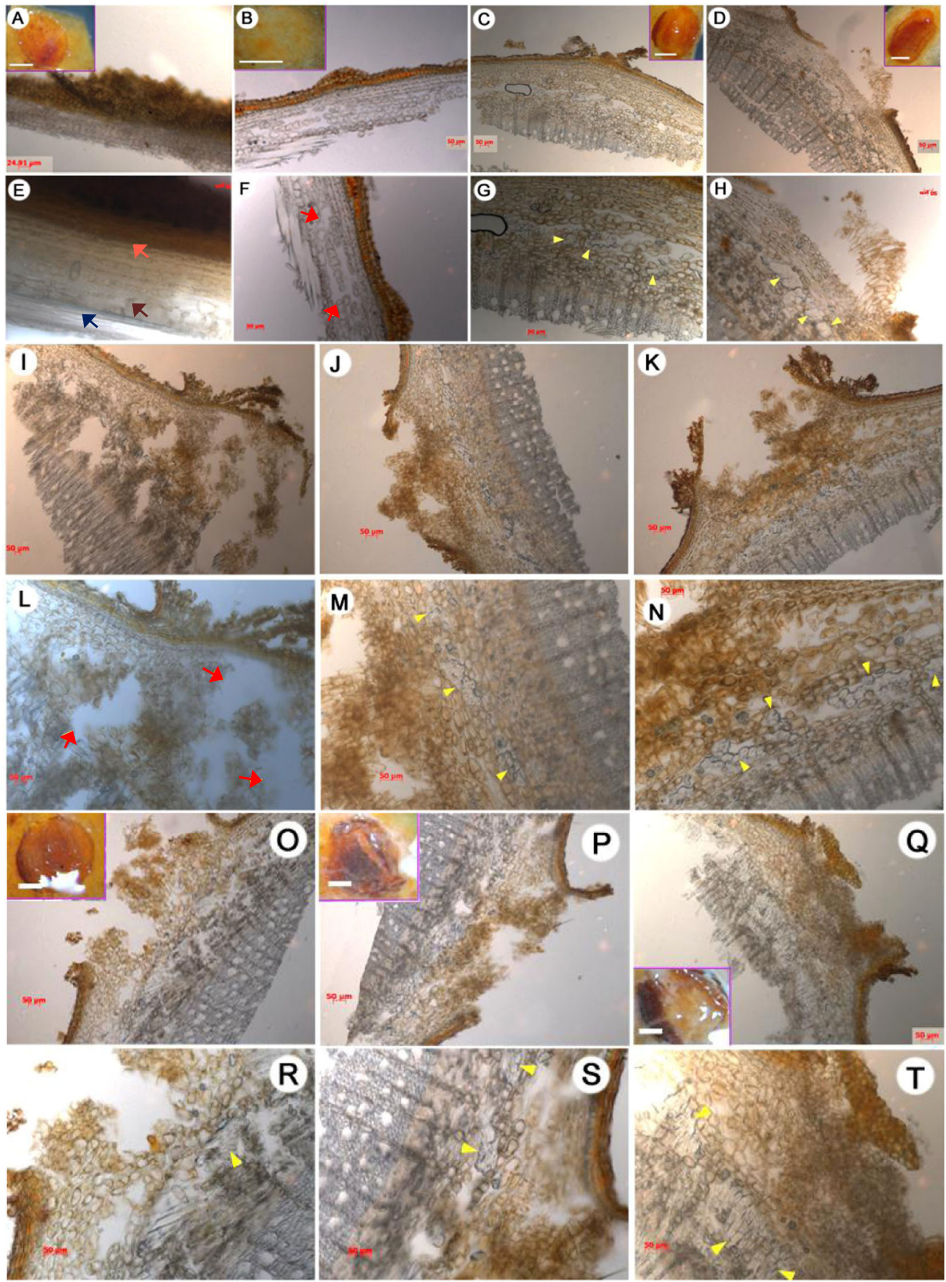
Anatomy of lenticels during the rooting of apple cuttings. Each lenticel was observed under a stereomicroscope at 0 h (**a**, **e**), 72 h (**b**–**d**, **f–h**), 120 h (**i–n**), and 168 h (**o**–**t**). The treatments are as follows: control (**a**, **e**, **c**, **g**, **j**, **m**, **p**, **s**), NPA (**b**, **f**, **i**, **l**, **o**, **r**), and IAA **(d**, **h**, **k**, **n**, **q**, **t**). Scale bars, 50 μm. Inset scale bars, **a**–**d** = **o**–**q** = 0.5 mm. Arrows: yellow, proliferated founder cells; pink, epidermis; brown, parenchyma cells located in the interfascicular cambium; dark blue, vascular tissues; red, cavities.

**Fig. 3:**
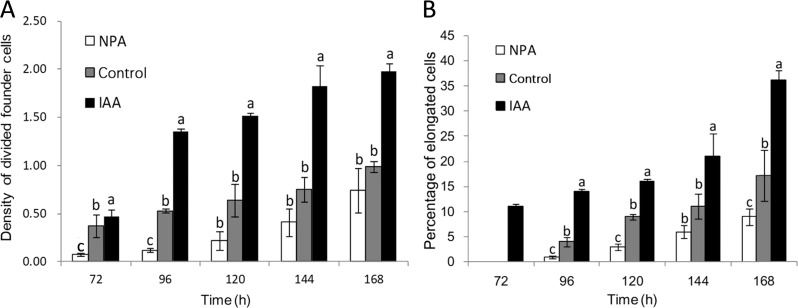
Density and elongation of founder cells during different AR developmental stages. **a** Density of divided founder cells in control, NPA-treated, and IAA-treated apple cuttings; the density is defined as the ratio of divided AR founder cells to the total number of parenchymal cells. **b** Percentage of elongated cells in control and NPA-treated and IAA-treated cuttings. The data are the percentage of the total elongated cells divided by number of founder cells that have undergone cell division. Elongated cells were those whose longitudinal axis was at least two times longer than the short axis. The error bar represents ±standard errors (SEs), *n* ≥ 15. The bars with different letters indicate statistical significance (*p* < 0.05).

The cells underlying the longitudinal splitting of the lenticels were examined to better understand the dehiscence process. The cambial zone in the control cuttings had 6–8 layers of cells between the xylem and phloem, and a large number of parenchymatous cells were observed in the interfascicular cambium next to the vascular cylinder (Fig. 2a, e; Fig. S1). From 72 to 168 h, founder cell divisions produced a large number of cells in the interfascicular cambium adjacent to the vascular tissues (Fig. 2a, c, e, g, j, m, p, and s). These founder cells exhibited dense cytoplasm and swollen nuclei (Fig. S2), features that are indicative of meristematic activity. Histological observations revealed differences in the founder cells underlying the lenticels in NPA-treated and IAA-treated cuttings compared with the control cuttings. Specifically, compared with those of the corresponding controls, the lenticels of the NPA-treated cuttings displayed fewer dividing founder cells at 72 and 168 h (Fig. 2b, f, i, l, o, and r). By contrast, the lenticels of IAA-treated apple cuttings had more founder and parenchymatous cells at 72 h (Fig. 2b–d, f–h) and more founder cells in the interfascicular cambium at 120 h than did the controls (Fig. 3a), and this process continued through 168 h (Fig. 2o, p, r, s). At 168 h, the number of elongated founder cells under lenticels continued to increase and started to protrude from the lenticel surface in IAA-treated apple cuttings (Fig. 2q, t). Together, these results indicate that lenticel dehiscence and AR protrusion begin by 72 and 168 h, respectively, and that IAA promotes lenticel longitudinal splitting during early AR emergence.

### Auxin controls founder cell division and elongation

To assess the situation of AR founder cell division at the very beginning of AR initiation, we defined the AR founder cell density as the ratio of divided AR founder cells to the total number of parenchymal cells. By 168 h, the density ratio of divided founder cells to total parenchymal cells was twofold greater in IAA-treated apple cuttings than in the controls (Fig. 3a). This time point also presented the highest number of divided founder cells in the IAA-treated cuttings (Fig. 3a). Taken together, these data suggest that IAA stimulates founder cell division. By contrast, the density of divided founder cells in the NPA-treated apple cuttings was approximately half of that of the control cuttings between 72 and 144 h (Fig. 3a); further, at 168 h, the density of divided founder cells in the NPA-treated apple cuttings was 0.75, comparable to that of the controls at 144 h (Fig. 3a). These observations suggest that NPA delayed founder cell division during late AR initiation. Overall, the density of divided founder cells increased over time in all conditions tested, with IAA and NPA treatments stimulating and inhibiting founder cell division, respectively.

Elongated founder cells were first observed at 72 h in the IAA-treated cuttings 24 h prior to their appearance in the controls (Fig. 3b). Interestingly, elongated founder cells were also observed at 96 h in the NPA-treated cuttings, but at a lower proportion compared to that in the control cuttings (Fig. 3b). Similar to that which occurred above, these data support the idea that IAA and NPA stimulate and inhibit, respectively, founder cell division. At 168 h, elongated founder cells accounted for 16.9%, 35.5%, and 8.8% of the total cells in the control, IAA-treated and NPA-treated cuttings, respectively. These data are consistent with the changes in divided founder cell density in the respective treatments from 96 to 168 h (Fig. 3a), suggesting that AR formation in apple is developmentally programmed.

### Organelle and endomembrane changes during AR initiation

**Fig. 4:**
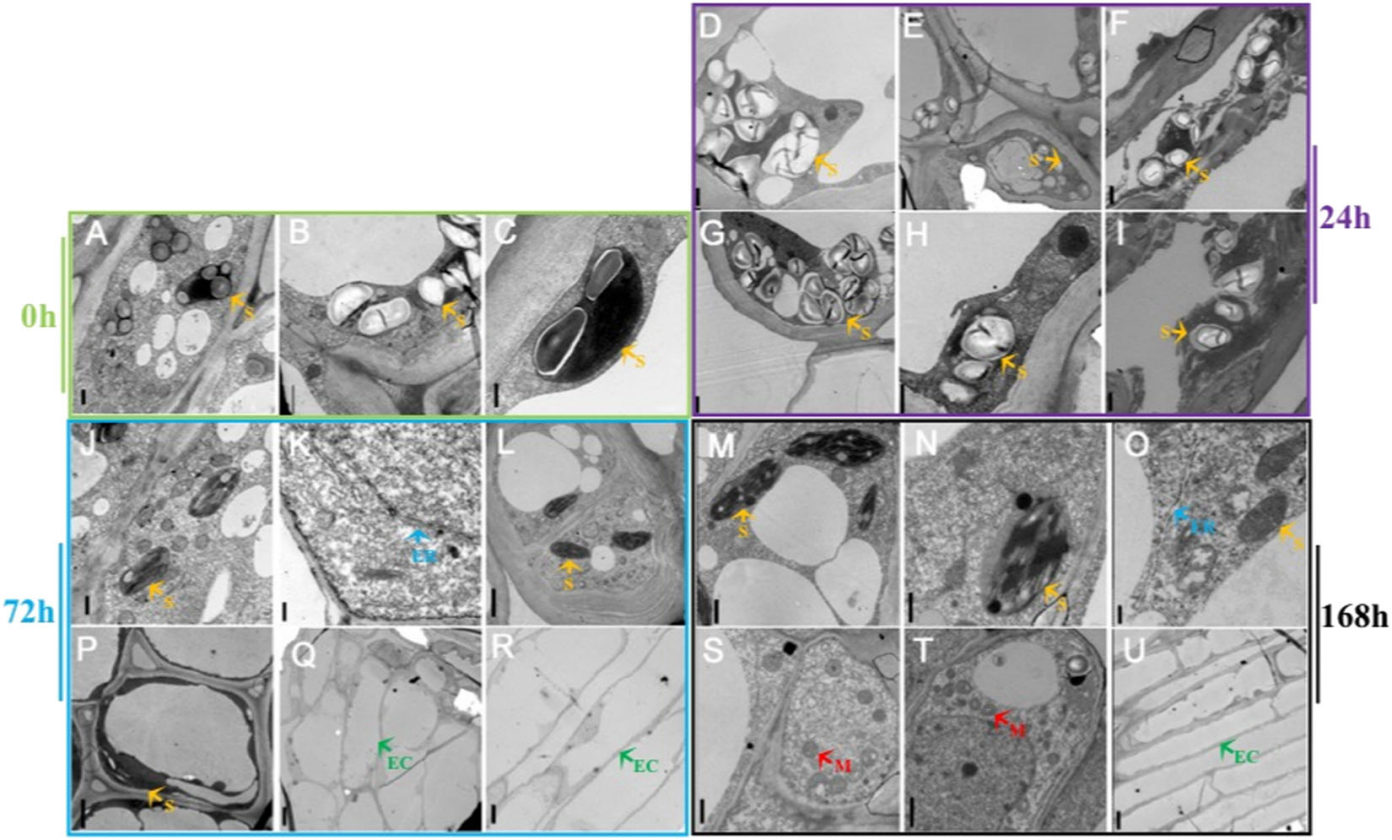
Ultrastructural changes during the rooting of apple cuttings. Transmission electron microscopy of founder cells at 0 h (**a**–**c**), 24 h (**d**–**i**), 72 h (**j**–**l**, **p**–**r**), and 168 h (**m**–**o**, **s**–**u**) during AR development. Shown are tissues from the control treatment (**a**–**c**, **e**, **h**, **k**, **q**, **n**, **t**), NPA treatment (**d**, **g**, **j**, **p**, **m**, **s**), and IAA treatment (**f**, **i**, **l**, **r**, **o**, **u**). Scale bars: 0.5 μm in **a**–**c**; 1.0 μm in **n**, **d**, **f**, **g–j**, **m, p**, **s** and **t**; 2.5 μm in **e** and **l**; 0.25 μm in **k** and **o**; and 5 μm in **q**, **r**, and **u**. Orange arrows, starch grains (**s**); blue arrows, endoplasmic reticulum (ER); green arrows, elongated cells (EC); red arrows, mitochondria (**m**).

To further investigate the morphological and physiological changes during AR initiation, subcellular structures were examined with transmission electron microscopy (TEM). Numerous starch grains were observed in the plastids at the 0 and 24 h time points for all treatments (Fig. 4a–i). However, at 72 h, the number and density of starch grains rapidly decreased from the plastids of control and IAA-treated cells, but little change in the density was observed in the plastids of the NPA-treated cells (Fig. 4j–l, p–r). At 72 h, no changes in starch grain content or organelles were observed in unsubmerged stems (Fig. S3).

The number and density of the mitochondria, endoplasmic reticulum, and Golgi apparatus in cells of IAA-treated cuttings were higher than those in the control cuttings at 72 h (Fig. S4). The number of elongated founder cells in the control and IAA-treated cuttings continued to increase from 72 h (Fig. 4q–r) to 168 h (Fig. 4m–o, s–u). By contrast, many dead cells and cells with abnormal nuclei were observed in the NPA-treated cuttings (Fig. S4). These observations, taken together with the near constant level of starch grains noted in the NPA-treated cuttings, suggest that NPA inhibits starch grain degradation, thus blocking the energy needed for founder cell division and elongation, resulting in abnormal subcellular structure and cell death.

### Auxin and cytokinin contents change in a complementary manner

**Fig. 5:**
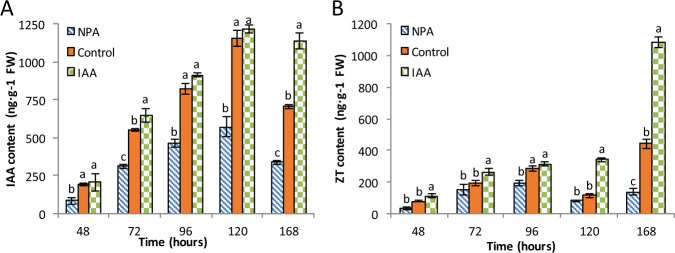
Indole acetic acid (IAA) and zeatin (trans-zeatin, ZT) levels during different developmental stages of rooting in apple cuttings. The error bar represents ±SEs, *n* ≥ 3. The different letters indicate significant differences (*p* < 0.05).

Both auxin and cytokinin are known to play key roles in AR initiation and elongation^5^. Therefore, we evaluated the changes in the concentrations of IAA and zeatin (trans-zeatin, ZT) during AR initiation in the control and IAA-treated and NPA-treated cuttings (Fig. 5). Although the IAA concentrations differed between the treatments, the trends in the changes in IAA levels were consistent between treatments during the test period. For example, the IAA concentration increased steadily with time from 48 to 120 h in all three treatments and peaked at 120 h at 1156.4 ng/g FW, 1216.6 ng/g FW, and 572.89 ng/g FW in the control, IAA and NPA treatments, respectively (Fig. 5a). This indicates that IAA increases during the whole AR formation process. At 168 h, the IAA levels in the control and NPA-treated cuttings decreased significantly to 707.2 ng/g FW and 338.8 ng/g FW, respectively, but decreased only slightly—to 1137.6 ng/g FW—in the IAA-treated cuttings compared with those at 120 h.

In the control and NPA-treated cuttings, ZT showed a three-phase accumulation pattern: it first increased, then decreased, and finally increased again by the end of the experimental period (Fig. 5b). In the control and NPA-treated cuttings, ZT levels increased from 48 to 96 h, decreased at 120 h, and increased again at 168 h (Fig. 5b). However, the decrease was not observed in the IAA-treated cuttings; instead, the ZT level was stagnant between time points 96 and 120 h, with a significant increase at 168 h. At 96 h, the ZT content in the IAA-treated samples was similar to that of the control. Overall, NPA-treated cuttings exhibited lower ZT levels compared to those in the control. For example, ZT levels in the NPA-treated cuttings were 36.84% and 72.50% lower than those in the control cuttings at 120 and 168 h, respectively (Fig. 5b). However, the control and NPA-treated cuttings exhibited a similar ZT accumulation pattern, which correlated well with that of the change in IAA levels (Fig. 5a). In contrast, ZT levels in the IAA-treated cuttings increased with time, with no decrease in concentration; in addition, the levels peaked at 1080.7 ng/g FW at 168 h (Fig. 5b), which was twofold that in the control cuttings at 168 h.

### *MdPIN* expression is stage-specific

**Fig. 6:**
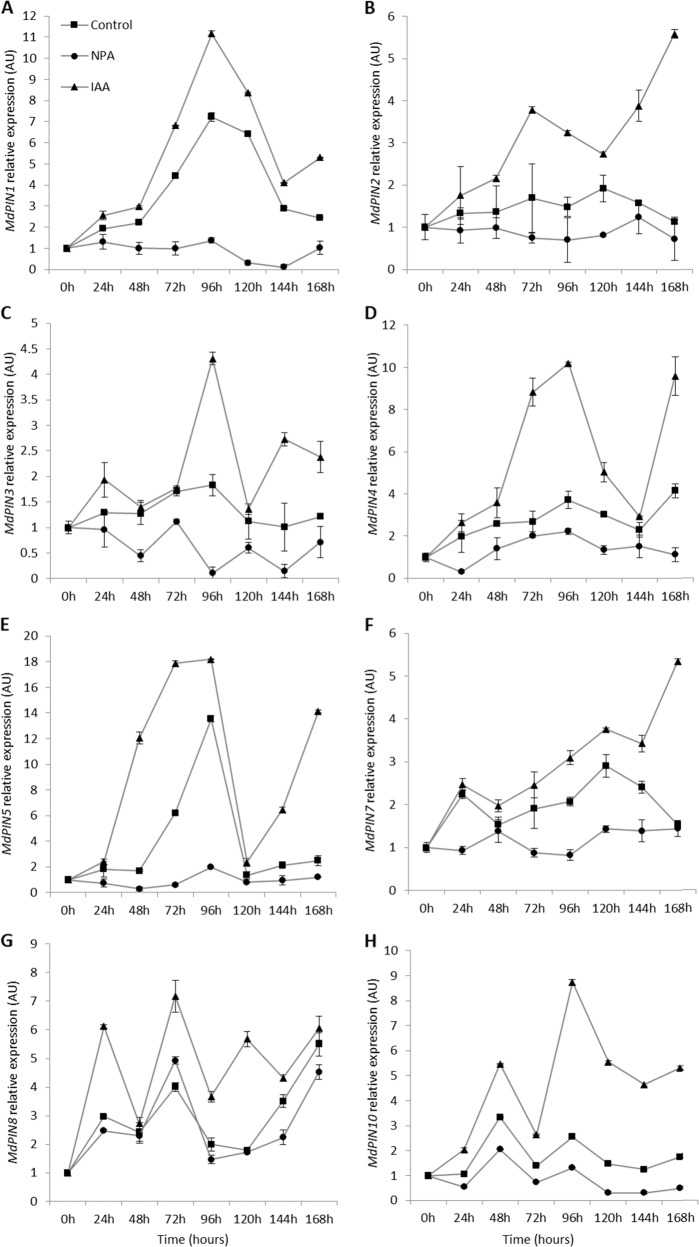
*MdPIN* gene expression. Gene expression levels of *MdPIN*s were quantified by qPCR in the bases of apple cuttings during different stages of AR formation. The average relative transcript level of each gene was calculated using the 2^−ΔΔCT^ method; the data are shown as the means ± SEs (*n* = 3).

To determine the correlations between *MdPIN* gene expression and the morphological and hormone changes associated with ARs in apple cuttings, we determined the transcript profiles of the eight *MdPIN* family members at each major stage of AR formation. All eight *MdPIN* genes were expressed during all three phases of AR formation but exhibited different spatiotemporal expression patterns (Fig. 6). For example, *MdPIN1* expression in the control cuttings increased from 0 h through 96 h and peaked at a level 7.2-fold that at 0 h. At 96 h, *MdPIN1* expression in the control and IAA-treated cuttings was 1.4-fold and 11.2-fold that at 0 h, respectively. In the NPA-treated and IAA-treated cuttings, *MdPIN1* expression showed the same decreasing trend at subsequent time points (Fig. 6a).

*MdPIN2* expression exhibited relatively uniform expression patterns across the time course, and the fold changes of *MdPIN2* expression between 24 and 168 h compared with the expression at 0 h ranged from 1.5 to 2-fold in the control and NPA-treated cuttings. *MdPIN2* expression was IAA responsive and increased by up to 5-fold from 24 to 168 h (Fig. 6b). NPA treatment seemed to decrease *MdPIN2* expression at all time points.

*MdPIN3* was expressed at relatively low levels during the time course (Fig. 6c). The expression levels peaked at 96 h in the control and IAA-treated cuttings and were approximately 1.8-fold and 4.3-fold that at 0 h, respectively. In addition, *MdPIN3* expression was inhibited by NPA at all time points, whereas IAA treatment stimulated *MdPIN3* expression, especially from 96 h until the subsequently time points (Fig. 6c).

In the control treatment, *MdPIN4* expression peaked at 96 and 168 h, with an approximately 4-fold increase compared with that at 0 h (Fig. 6d). *MdPIN4* expression appeared to be sensitive to IAA and NPA treatments after 24 h, with the highest expression levels occurring at 96 h, which were 2.2-fold and 10.2-fold higher than the levels at 0 h in the NPA-treated and IAA-treated cuttings, respectively.

*MdPIN5* had the highest level of expression in the control treatment at 72 h and 96 h, with a 13.5-fold increase at 96 h in comparison to that at 0 h (Fig. 6e). *MdPIN5* expression was IAA sensitive, and its expression increased in accordance with the same pattern as that of the controls, with an increase of 18.2-fold at 96 h. NPA treatment inhibited *MdPIN5* expression at all time points.

*MdPIN7* expression in the control peaked at 120 h, reaching 3.6-fold that at 0 h (Fig. 6f). *MdPIN7* expression was sensitive to IAA starting at 72 h, and the expression increased to 5.3-fold at 168 h. *MdPIN7* showed a weak response to NPA treatment.

*MdPIN8* expression increased 3-fold at 24 h compared to the baseline expression level at 0 h, and IAA treatment-induced *MdPIN8* expression during the time course (Fig. 6g). In contrast, NPA slightly inhibited *MdPIN8* expression. Relatively high expression of *MdPIN8* occurred at 72 and 168 h, corresponding to the initiation phase. *MdPIN8* expression in the control peaked at 168 h, reaching 5.5 times that at 0 h. In NPA-treated and IAA-treated cuttings, *MdPIN8* expression peaked at 72 h, reaching 4.9-fold and 7.2-fold that at 0 h, respectively. Furthermore, NPA treatment increased *MdPIN8* expression at 72 and 168 h.

*MdPIN10* in the IAA-treated cuttings was also expressed during the early stages of AR formation, peaking at 48 h—2-fold above baseline. At 48 h, *MdPIN10* expression in the control and IAA-treated cuttings increased by 3.4-fold and 5.6-fold, respectively, compared with the levels at 0 h (Fig. 6h). In the IAA-treated cuttings, *MdPIN10* expression peaked at 8.6-fold at 96 h. In contrast to *MdPIN8* expression, *MdPIN10* expression tended to be inhibited by NPA at all time points.

## Discussion

### AR formation requires the initiation of new founder cells from the interfascicular cambium adjacent to vascular tissues

AR formation is crucial for commercial propagation, and depending on the species, there are two mechanisms for AR formation in most woody plants. AR founder cells can either (1) initiate in the stem but remain dormant until the induction of AR formation by environmental conditions^5^ or (2) initiate de novo from cells, such as phloem or xylem parenchyma cells, within or adjacent to vascular tissues, such as cells of the interfascicular cambium or the phloem/cambium junction^3,5,23^. A typical apple stem contains collateral vascular bundles with a ring around the pith (Figs. 1 and 2, Fig. S1). Initially (at 0 h), the cells underlying the lenticels in the interfascicular cambium adjacent to vascular tissues had no observable meristematic activity, whereas the founder cells had already undergone numerous cell divisions 72–168 h post-cutting (Figs. 2 and S2). Therefore, AR formation in apple rootstock M.9 cuttings occurs via the second mechanism. This is consistent with recently reported findings in forest tree species, especially conifers, in which roots are induced from determined or differentiated cells and further from positions where roots do not normally occur during development^24^.

### ARs protrude through lenticels

Lenticel formation culminates with the disintegration of cork cells under the cork cambium; these cork cells are ultimately replaced by loosely arranged thin-walled cells, also called supplemental cells or filling cells, during early cork layer formation. Later, the epidermis and cork are squeezed until they crack as supplemental cells continue to divide and eventually split into a series of lip projections, which constitute lenticels^25^. The tissues inside lenticels provide a natural channel not only for gas exchange during flooding or other abiotic stresses but also AR formation and growth. In this study, the lenticels of apple cuttings exhibited an increasingly larger crack during the early stage of AR formation (Fig. 1a–g). Our results showed that, at each time point during AR development, the lenticels of control and IAA-treated cuttings were more severely ruptured by founder cell division compared with those of the NPA-treated cuttings (Fig. 1h–u). These developmental changes indicate that auxin promotes lenticel dehiscence and AR protrusion from lenticel channels. This process is similar to auxin-mediated channel formation for LR emergence in *A. thaliana*^26,27^. In both cases, auxin uptake into cortical cells and epidermal cells overlying the LR primordium during emergence results in increased expression of genes encoding cell wall-remodeling enzymes, such as pectate lyase, to promote cell separation for LR emergence^26,27^. In addition, histological observations revealed intrastem channels formed by enlarged cavities in the interfascicular cambium, and the number and dimensions of these cavities increased over time (Fig. 2). These results further suggest that the cells located in front of newly formed founder cells might have undergone programmed cell death (PCD) to allow AR primordium development, supporting the conclusion of a previous study on AR formation in tomato^28^.

### Induction phase of AR formation in apple cuttings

AR formation in apple cuttings can be divided into three phases based on characteristic cellular changes as described in the anatomical observations: induction (0–72 h), initiation (72–120 h), and extension (120–168 h). In the control and IAA-treated cuttings, the induction phase at 72 h was characterized by an increased number of founder cells with dense cytoplasm and swallow nuclei (Fig. 2g, h), as well as hydrolysis of starch grains (Fig. 3p, q), which presumably provided energy for the further division and elongation of AR founder cells. By contrast, a large number of starch grains remained in the chloroplasts at 72 h in NPA-treated apple cuttings (Fig. 4j), suggesting that NPA inhibited starch hydrolysis during AR formation, whereas IAA enhanced the conversion of starch into “cash energy” for AR induction. These findings suggest that IAA modulates AR induction by inducing the dedifferentiation of interfascicular cambial cells into AR founder cells.

Thus, AR initiation begins with the division and elongation of a large number of clustered founder cells that filled the cavity opened by lenticel splitting in the control and IAA-treated cuttings (Figs. 2m, n and 3f–n), whereas founder cell division was severely impaired in the NPA-treated apple cuttings (Fig. 2l). In addition, IAA treatment significantly increased the ratio of divided founder cells to the total number of parenchymal cells, and founder cell density increased more quickly in these cuttings than in the controls (Fig. 3a). These data demonstrate the promoting effect of IAA on primordial founder cell division upon the induction of AR formation (Fig. 3a).

With elongation of the founder cells, AR formation progresses to the extension phase when the percentage of elongated cells out of the number of divided founder cells increases (Fig. 4p, q, s, t). Elongated AR founder cells occurred earlier in the IAA-treated cuttings and were almost twice as abundant as those in control cuttings (Fig. 3b). Thus, we speculate that both lenticel dehiscence and intrastem PCD^28^ might regulate AR formation by modulating PAT, which regulates the auxin gradient.

### Starch grain depletion is associated with AR initiation

Previous studies have found that maintaining an appropriate auxin level and gradient in the basal portion of shoots is essential for AR formation^17,29,30^, but the exact mechanism underlying this phenomenon is still unclear. Here, we observed rapid starch grain depletion upon the initiation of AR formation in apple cuttings. It has been proposed that starch accumulation and depletion may be a biochemical indicator of early root formation in hypocotyl cuttings, which provides energy for AR formation^31^. This energy supplementation process is positively regulated by auxin^32–34^. In agreement with the published data, the results of our temporal and spatial analyses studies also support a key role of starch grain accumulation and degradation in AR formation in woody plants, with IAA exerting a stimulatory effect on AR formation and NPA delaying this process (Fig. 4). In addition, starch grain depletion was also associated with endomembrane proliferation and reorganization (Figs. 4 and S4). Although the physiological basis of how these two processes relate to AR formation is known, the fact that grain depletion and new membrane system formation were promoted by IAA and inhibited by NPA suggests that PAT is the initial driving force for these processes^35^.

### Roles of auxin and cytokinin during AR formation

Recent studies have shown that ZT can suppress AR primordium formation, whereas auxin signaling positively regulates this complex biological process, suggesting that relatively high IAA and low ZT levels are prerequisites for AR induction and initiation^36,37^. Cytokinins and auxin seem to exert opposite effects on AR formation^4,38^. In addition, auxin can directly downregulate cytokinin biosynthesis, while cytokinins have little effect on auxin biosynthesis^39^. The initial high levels of auxin in cuttings during the induction and initiation phases of AR formation were replaced by high levels of cytokinin during the extension phase, suggesting that crosstalk occurs between auxin and cytokinin metabolism, although the molecular basis remains to be characterized.

Auxin appears to regulate AR formation mainly by modulating AR founder cell division and elongation during the induction and initiation stages (Figs. 2 and 3). Similar to that observed for LR formation in *A. thaliana*^40^, IAA levels in apple cuttings continued to increase during the induction (0–72 h) and initiation (72–120 h) phases, peaking at 120 h, followed by a steady reduction after entering the elongation phase (120–168 h). ZT levels also increased during the induction and initiation phases and peaked at 96 h before they started to decrease. By contrast, NPA reduced IAA accumulation but had little effect on ZT levels. These results point to the maintenance of auxin and cytokinin homeostasis via feedback regulation. Recent studies monitoring the functions between auxin and cytokinin during the apple cutting rooting process showed that the ratio of auxin to cytokinin also increased during the AR induction phase, which may be due to the occurrence of cellular programming and the need for cell division^36^. Further molecular data supported that alterations in CTK levels may function as feedback in regulating the expression of auxin-related genes such as *SHY2* and *PIN1*^36^.

### Correlations between *MdPIN* expression and AR formation

**Fig. 7:**
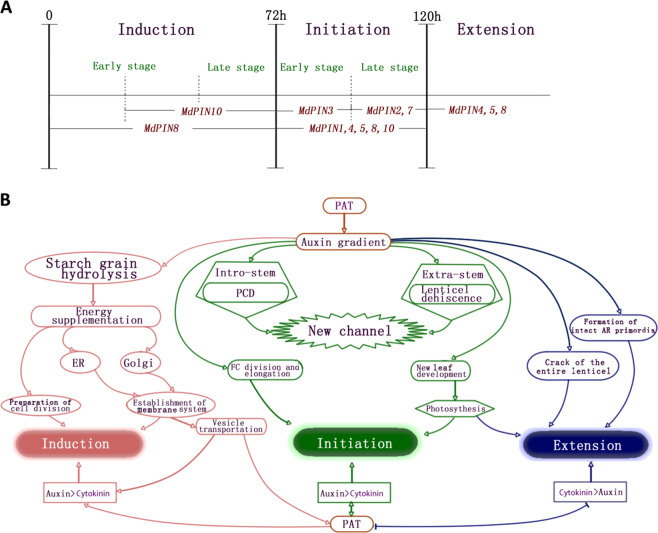
Stage-specific *MdPIN* gene expression patterns and proposed model of cellular regulatory mechanisms during AR formation. **a** Differential expression of *MdPIN*s during AR induction, initiation, and extension. The dashed lines represent 24 h time points. *MdPIN8* expression peaked during the induction phase (24 h), indicating that *MdPIN8* may play a major role in the early stage of AR induction. *MdPIN10* expression peaked at 48 h and was mostly low at the other time points, suggesting that *MdPIN10* also contributes to the induction and initiation of ARs. The maximum expression of *MdPIN1*, *MdPIN3*, *MdPIN4*, and *MdPIN5* occurred at 96 h, just 24 h prior to the morphological changes in the initiation phase, which ended at 120 h. Further, *MdPIN1*, *MdPIN2*, *MdPIN4*, and *MdPIN5* expression remained high at 120 h, indicating that all of these genes also function during the late stage of initiation. *MdPIN7* expression peaked at 120 h, suggesting that *MdPIN7* mainly functions during the late stage of initiation. At 168 h, differential increases in *MdPIN4*, *MdPIN5*, and *MdPIN8* expression were observed, suggesting that these members promote lenticel dehiscence and AR protrusion from the epidermis during the extension phase. **b** Proposed model of cellular regulatory mechanisms during AR formation stages. ER endoplasmic reticulum, PAT polar auxin transport, PCD programmed cell death. The arrows represent positive regulatory elements. The lines ending with a bar represent negative regulatory elements.

Most *PIN*s function in directional auxin transport while displaying differential expression patterns, reflecting their potential multifaceted roles in plant development^41–43^. In Arabidopsis, members of the *PIN* gene family participate in vascular PAT, root patterning, root gravitropism, sink-driven auxin gradient establishment, and apical–basal polarity^44–49^. The *PIN* family in apple comprises eight members, all of which are expressed differentially during AR formation (Fig. 6). Therefore, we proposed a possible model of the regulatory mechanism to map the main function of each *MdPIN* during the rooting of apple cuttings (Fig. 7a).

Upregulation of *MdPIN8* and *MdPIN10* was mainly associated with AR induction. The AR initiation phase seems associated with an upregulation of *MdPIN1*, *MdPIN4*, *MdPIN5*, *MdPIN8*, and *MdPIN10*; *MdPIN3* was upregulated at the early stage of initiation, whereas *MdPIN2* and *MdPIN7* were upregulated toward the end of the initiation stage (Fig. 7a). The extension phase is mainly associated with upregulation of *MdPIN4*, *MdPIN5*, and *MdPIN8* (Fig. 7a). Our data suggest that different *MdPIN*s likely participate in the different phases of the AR formation process in a unique way; for example, their gene products may have directly or indirectly regulate AR formation in a cooperative manner by mediating anatomical and physiological changes in the cuttings (Fig. 7b). Future biochemical studies should focus on the diverse expression patterns of *MdPIN* proteins and employ in situ hybridization tests to further investigate the function of each in regulating this postembryonic rooting process.

## Conclusions

AR formation in apple is a coordinated developmental process. In our study, upregulation of *PIN* gene expression seems to be the result of IAA stimulation during the AR initiation phase of apple. IAA stimulates AR founder cell division and elongation probably by promoting energy supplementation via starch grain hydrolysis, which leads to endomembrane system proliferation, lenticel dehiscence, and AR emergence in the apple M.9 rootstock.

## Materials and methods

### Plant material and growth conditions

Six-month-old apple M.9 cuttings were used for all experiments. The bases of the cuttings (0–4 cm) were submerged in ventilated hydroponic containers filled with Hoagland’s nutrient solution (pH 5.8)^50^, and allowed to grow in a growth chamber (~25 °C) under a 16:8 L/D cycle under a light intensity of 300 μmol m^−2^ s^−1^. All plant samples were collected at 10:00 a.m. each day and were harvested every 24 h for 168 h (7 days). Plant materials used for morphological studies were fixed in 2.5% glutaraldehyde, and those used for phytohormone quantitation and gene expression analysis were frozen in liquid nitrogen and then stored at −80 °C until further use.

### Treatments and growth analysis

Plants were grown in Hoagland’s nutrient solution (control) or nutrient solution supplemented with 10 μM indole-3-acetic acid (IAA) or N-1-naphthylphthalamic acid (NPA) as positive and negative regulators of PAT, respectively. The IAA and NPA were purchased from Sigma-Aldrich (location is China, catalog numbers 12886 and 33371, respectively) and dissolved in dimethyl sulfoxide.

### Anatomical and ultrastructural observations

Cryosectioning was used to observe anatomical changes during AR initiation. Samples were dehydrated and fixed in 2.5% glutaraldehyde solution according to the methods of Chen et al.^51^. The division and elongation of AR founder cells were observed with a stereoscopic microscope (Leica MZ 6; Wetzlar, Germany), and ImageJ (v. IJ 1.46r) was used for data capture and analysis^52^. Statistical differences in AR density and percent elongation were determined by ANOVA in conjunction with Tukey’s post hoc test in SPSS 20^53^, at *p* < 0.05. Cryosectional samples were also used for SEM and TEM.

### Quantitation of free phytohormones

Submerged apple cuttings with approximately equal weight, length, and diameter were collected for each treatment, and the location 5 mm above the incision of the cuttings was used to determine IAA and ZT contents. The samples were incubated in ice-cold uptake buffer (1.5% sucrose, 23 mM MES; pH 5.5) for 15 min, followed by two 15-min washes with fresh uptake buffer. The cleared tissue was surface dried on filter paper and then weighed. The IAA and ZT levels were measured using HPLC–ESI–MS/MS^54^. The reverse-phase HPLC gradient parameters and selected reaction monitoring conditions for protonated or deprotonated plant hormones ([M+H]+ or [M−H]−) are listed in Supplementary Tables S1 and S2. IAA and ZT standards were purchased from Sigma (catalog numbers 12886 and Z0876, respectively).

### RNA extraction and qPCR

RNA was extracted using the CTAB method, and 1 μL was used as a template for cDNA synthesis using a TaKaRa PrimeScript RT Reagent Kit (RR037B; Liaoning, China). Quantitative PCR (qPCR) was performed with an ABI Prism 7900HT system (Applied Biosystems; Foster City, CA, USA) using a 10-fold dilution of the cDNA template. The naming of MdPIN family genes based on homology identification in Arabidopsis and the gene ID of each *MdPIN* are shown in Supplementary Table 3. The reaction mixture included 5 μL of template, 7.5 μL of SYBR Green PCR master mix (4309155; Applied Biosystems), and the two *MdPIN*-specific primers at 1 μM each (Table S4) in a final volume of 15 μL. Primer efficiency was determined with a standard curve analysis using a 5-fold serial dilution of a known amount of template, and amplicon specificity was confirmed by sequencing. The thermal cycler regime was as follows: 2 min at 50 °C; 10 min at 95 °C; and then 40 cycles of 15 s at 95 °C, 30 s at 54 °C, and 30 s at 72 °C. Disassociation curves and gel electrophoresis were used to verify the amplification of a single product. *C*_T_ values were calculated using SDS 2.1 software (Applied Biosystems), and the data were analyzed using the 2^–ΔΔCT^ method, with 18S rDNA serving as a reference gene for normalization^55^.

## Supplementary information

Supplemental data
