# Supplementary material for: Auxin regulation and MdPIN expression during adventitious root initiation in apple cuttings
Source: Hortic Res. 2020 Sep 1;7:143. doi: 10.1038/s41438-020-00364-3 (PMC7459121; doi:10.1038/s41438-020-00364-3)
Supplement: Supplementary file 1 — Supplemental data [file 41438_2020_364_MOESM1_ESM.doc]

**Figure S1.** Lenticel tissues in apple cutting at 0h. Orange arrows point to grains inside of the parenchyma cells, which are located among interfascicular cambium; red and dark blue arrows point to epidermis and vascular tissues, respectively. Bar, 150 μm.


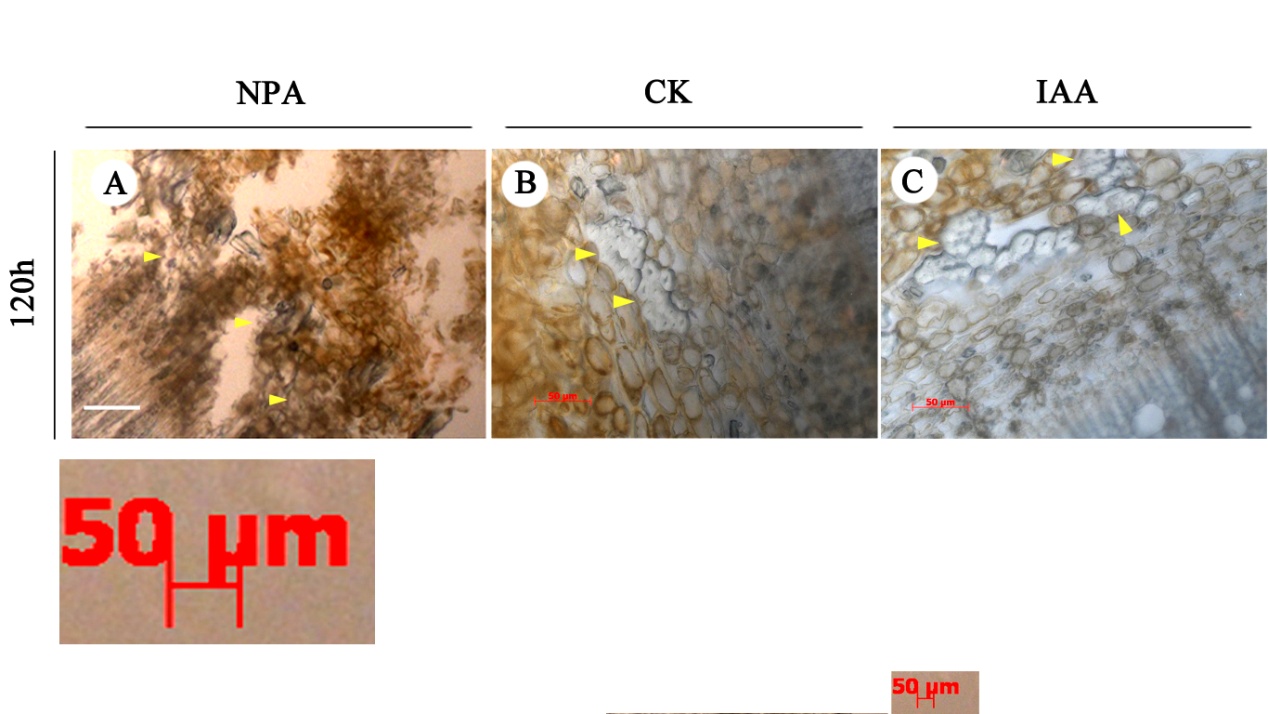


**Figure S2**．Divided founder cells are located in the cambium, and exhibit dense cytoplasm and enlarged nuclei. Yellow arrows point to divided founder cells. Bar, 50 μm.

**Figure S3.** Transmission electron microscopy of an unsubmerged apple cutting at 72h. Neither the starch grains (yellow arrows) reduction nor organellar changes (blue, endoplasmic reticulum (ER); red, mitochondria (M)) were observed in control cuttings (CK) treated with IAA or NPA compared to submerged tissue. Bar, A, C, E=2.5μm; B, D=1.0μm; F=5.0μm.

**Figure S4** Transmission electron microscopy of cells around lenticels of submerged cuttings at 72h. A. Abnormal nuclear morphology (yellow arrow) and cell death at 72h in NPA-treated apple cutting. B. Mitochondria (red arrow) frequency was significantly increased while the number of starch grains was reduced in control cells, further indicating that the starch grains maybe hydrolyzed and released as an energy source for mitochondria to provide “cash energy” during AR formation. C. Endoplasmic reticulum (arrow blue) and Golgi (arrow purple) appearance frequency increased, whereas the starch grains almost diminished (arrow orange), revealed an endomembrane system building process in 72h IAA treatment of apple cutting rooting. Bar, A=2.5 μm, B=C=0.5μm.

**Supplemental Table 1**. Reverse-phase high-performance liquid chromatography (HPLC) gradient parameters in mobile phases

| **Time (min)** | **Gradient* (volume percentage)** |
| --- | --- |
| **0-2** | 30 |
| **2-20** | Increase linearly to 100 |
| **20-22** | 100 |
| **22-25** | Decrease linearly to 30 |

*****The binary solvent system uses water with 0.1% (vol/vol) formic acid and methanol with 0.1% (vol/vol) formic acid as mobile phases. Separations were performed by altering the percentage of mobile phases.

**Supplemental Table 2**. Selected ion reaction monitoring conditions for protonated or deprotonated plant hormones of IAA and ZT ([M + H]^+^ or [M − H]^−^ ).

| **PH** | **SM** | **Q1** | **Q3** | **Q2(V)** | **RT*** |
| --- | --- | --- | --- | --- | --- |
| **IAA** | - | 174.0 | 129.6 | -14 | 5.22 |
| **ZT** | + | 220.2 | 136.2 | 29 | 2.91 |

IAA, indole-3-acetic acid; ZT, zeatin; Q1, precursor ion selected in Q1; Q3, product ion selected in Q3; Q2, collision energy (V); SM, scan mode.

*RT (retention time) listed in this table are obtained under HPLC and column conditions mentioned in the method.

**Supplemental Table 3**. List of PIN genes found in apple (*Malus x domestica*), gene accessions were retrieved from the Genome Database for Rosaceae (GDR: <http://www.rosaceae.org/>).

| **Gene name** | **Lg** | **Gene ID** | **Genbank ID** | **Position (Mb)** |
| --- | --- | --- | --- | --- |
| **MdPIN1** | **14** | **MDP0000138035** | **EF406255** | **28.405** |
| **MdPIN2** | **-** | **MDP0000119864** | - | **-** |
| **MdPIN3** | **1** | **MDP0000156440** | **-** | **2.567** |
| **MdPIN4** | **16** | **MDP0000234528** | **EF406257** | **0.183** |
| **MdPIN5** | **4** | **MDP0000297331** | **-** | **19.946** |
| **MdPIN7** | **13** | **MDP0000497581** | **EF406258** | **1.552** |
| **MdPIN8** | **9** | **MDP0000250518** | **-** | **2.797** |
| **MdPIN10** | **6** | **MDP0000200231** | **EF406260** | **23.865** |

**Supplemental Table 4.** Primer sequences used for quantitative real time PCR analysis of *MdPIN* genes expression during rooting in apple cuttings.

| **Gene** | **Forward Primer (5' - 3')** | **Reverse Primer (5' - 3')** |
| --- | --- | --- |
| ***MdPIN1*** | CACTCTGCCTAACACTCTG | CTTGCTCCTCGGTATTCG |
| ***MdPIN2*** | GCTGTGTGGGTGAAACTT | CACTGCTGCCTGAATGAT |
| ***MdPIN3*** | GTTGGACTGCGAGGTGTT | GGTGCTTAGTATGTCAGGATG |
| ***MdPIN4*** | GCAATGGCAGTAAGGTTC | TGGACAATCGCTAAGTGG |
| ***MdPIN5*** | TACAAGGTGGTGGTAGCC | CGATGAAGCGGTAGTTCC |
| ***MdPIN7*** | ATCCGTTGATCTTGTTGTTC | TCTATGTCCTCCCCTTGTG |
| ***MdPIN8*** | GCGTTCCAATCCCAAATC | GTTCCTGAAGAAGTGATAGC |
| ***MdPIN10*** | ACGCTTGTTTACTACATTTTG | TATGAGCTTCTTGCTTTCTG |
| ***18S*** | GTTACTTTTAGGACTCCGCC | TTCCTTTAAGTTTCAGCCTTG |
